# Mass-flowering crops enhance wild bee abundance

**DOI:** 10.1007/s00442-012-2515-5

**Published:** 2012-11-01

**Authors:** Andrea Holzschuh, Carsten F. Dormann, Teja Tscharntke, Ingolf Steffan-Dewenter

**Affiliations:** 1 Department of Animal Ecology and Tropical Biology, Biocenter, University of Würzburg, Am Hubland, 97074 Würzburg, Germany; 2 Biometry and Environmental System Analysis, University of Freiburg, Tennenbacher Str. 4, 79106 Freiburg, Germany; 3 Agroecology, Georg-August University, Grisebachstr. 6, 37077 Göttingen, Germany

**Keywords:** Canola, Oilseed rape, Pollen, Spillover, Trap nests

## Abstract

Although agricultural habitats can provide enormous amounts of food resources for pollinator species, links between agricultural and (semi-)natural habitats through dispersal and foraging movements have hardly been studied. In 67 study sites, we assessed the interactions between mass-flowering oilseed rape fields and semi-natural grasslands at different spatial scales, and their effects on the number of brood cells of a solitary cavity-nesting bee. The probability that the bee *Osmia bicornis* colonized trap nests in oilseed rape fields increased from 12 to 59 % when grassland was nearby, compared to fields isolated from grassland. In grasslands, the number of brood cells of *O. bicornis* in trap nests was 55 % higher when adjacent to oilseed rape compared to isolated grasslands. The percentage of oilseed rape pollen in the larval food was higher in oilseed rape fields and grasslands adjacent to oilseed rape than in isolated grasslands. In both oilseed rape fields and grasslands, the number of brood cells was positively correlated with the percentage of oilseed rape pollen in the larval food. We show that mass-flowering agricultural habitats—even when they are intensively managed—can strongly enhance the abundance of a solitary bee species nesting in nearby semi-natural habitats. Our results suggest that positive effects of agricultural habitats have been underestimated and might be very common (at least) for generalist species in landscapes consisting of a mixture of agricultural and semi-natural habitats. These effects might also have—so far overlooked—implications for interspecific competition and mutualistic interactions in semi-natural habitats.

**Electronic supplementary material:**

The online version of this article (doi:10.1007/s00442-012-2515-5) contains supplementary material, which is available to authorized users.

## Introduction

Intensive agriculture has caused alarming declines in farmland biodiversity (Krebs et al. 1999). One of the main reasons for the negative effects of agriculture is the expansion of agricultural land at the cost of semi-natural and natural habitats (henceforth termed natural habitats). However, intensively used agricultural landscapes often provide resources even for species that depend on natural habitat at least temporally during their life cycle (Dunning et al. 1992). Many of these species are responsible for important ecosystem services such as biocontrol and pollination in both agricultural and natural habitats (Aguilar et al. 2006; Rand and Louda 2006; Klein et al. 2007; Letourneau et al. 2009). The presence of species in agricultural habitats may indicate a benefit from resources provided there. While cross-habitat spillover from natural habitats on agricultural habitats has been relatively well documented, there is a general lack of studies addressing effects of agricultural on natural habitats (Blitzer et al. 2012).

Managed habitats often surpass natural habitats in the amount of food resources they offer, such as plant biomass attracting herbivores and subsequently their predators (Rand et al. 2006), or the amount of pollen- and nectar-attracting pollinators (Morandin and Winston 2005; Carvalheiro et al. 2011). Mass-flowering oilseed rape fields provide 350,000–700,000 plants per hectare, each producing more than 100 flowers (Hoyle et al. 2007) during a flowering period of about 4 weeks. Because of the enormous food density and the easy accessibility of nectar and pollen, foraging on mass-flowering crops like oilseed rape may pay off for wild bees from nearby natural habitats, despite having to fly back to their nesting sites after every foraging trip.

Besides the decline of nesting sites, which are almost exclusively found in natural habitats, the decline of food resources is supposed to be the major threat of wild bees in many regions worldwide (Holzschuh et al. 2008; Steffan-Dewenter and Schiele 2008; Potts et al. 2010). While, up to now, most research has focused on the negative effects of increased cover of agricultural land and the simultaneous decrease in natural habitats (e.g., Ricketts et al. 2008; Garibaldi et al. 2011), potential resource support from agricultural land for populations in natural habitats has been disregarded (Rand et al. 2006; Blitzer et al. 2012). Cross-habitat fluxes of individuals and food resources from agricultural land to natural habitats can be expected to have often substantial consequences for the fitness of individuals reproducing in natural habitats, and subsequently for population dynamics, species interactions and ecosystem services in both natural and agricultural habitats (Diekötter et al. 2010; Gladbach et al. 2011; Holzschuh et al. 2011; Jauker et al. 2012).

The area cultivated with mass-flowering oilseeds in Europe has increased by 49.9 % between 2000 and 2010 (European Commission 2011), largely due to an increased demand for biofuel during the last decade. However, the impact of oilseed rape on solitary wild bees has so far hardly been studied. Related studies from social bumblebees that were conducted several weeks after the flowering period of oilseed rape showed positive effects of oilseed rape on abundances of short-tongued bumblebees (Westphal et al. 2006; Diekötter et al. 2010), mixed effects on abundances of long-tongued bumblebees (Westphal et al. 2006; Diekötter et al. 2010), and no effects on the percentage of colonies producing sexual offspring (Westphal et al. 2009). Studies on solitary bees show that high amounts of oilseed rape at the landscape scale have positive effects on a solitary bee nesting in semi-natural habitats (Jauker et al. 2012), and suggest that flowering oilseed rape can counteract negative effects of low wild flower densities in nearby semi-natural habitats (Holzschuh et al. 2011). Jauker et al. (2012) proposed as a possible explanation for these positive effects that bees benefit from the abundant nectar provided by oilseed rape, and claimed the equally abundant pollen supply not to be important. However, a study on the impact of oilseed rape pollen on the abundance of bees nesting in semi-natural habitats is so far lacking.

In our study, we focused on the impact of oilseed rape on the abundance of the solitary and polylectic Red Mason Bee *Osmia bicornis* L. (Megachilidae), which nests in cavities in natural and semi-natural habitats. In 67 study sites, we assessed the effects of oilseed rape on bees in semi-natural grasslands and vice versa at the local scale (habitat types directly adjoining vs. isolated habitats) and at the landscape scale (low to high proportions of oilseed rape or semi-natural habitats). In artificial nests, we evaluated the number of brood cells and the percentage oilseed rape pollen in larval food as well as the relationship between the percentage oilseed rape pollen and the number of brood cells.

We hypothesized that

1. without nearby source habitat, *O. bicornis* will not nest in oilseed rape fields; i.e., the occurrence of *O. bicornis* in trap nests in oilseed rape fields increases when grassland adjoins;
2. *O. bicornis* profits from mass-flowering oilseed rape; i.e., the number of *O. bicornis* brood cells in trap nests in grasslands adjacent to oilseed rape is higher than in isolated nesting aids and increases with the proportion of oilseed rape in the landscape;
3. the percentage oilseed rape pollen in larval food increases with local availability (highest in oilseed rape fields, intermediate in grasslands adjacent to oilseed rape and lowest in grasslands isolated from oilseed rape and with landscape-scale availability of oilseed rape pollen (i.e. proportion of oilseed rape fields in the landscape);
4. oilseed rape pollen contributes substantially to offspring provisioning by *O. bicornis*, leading to increases in the number of brood cells with increasing percentage of oilseed rape pollen in larval food; additionally, the positive impact of oilseed rape pollen should decrease with increasing availability of alternative food resources at the local and landscape scale.

## Materials and methods

### Study sites

The study was carried out in 2007 near the city of Göttingen (51.5°N, 9.9°E), Lower Saxony, Germany. In an area of about 25 × 30 km, we selected 67 study sites (33 calcareous grasslands and 34 oilseed rape fields belonging to four categories (ESM 1): (1) 16 grasslands were isolated by at least 230 m from the nearest oilseed rape field (mean distance from grassland to field edge ± SE: 481 ± 8.8 m); (2) 17 grasslands were within 1–15 m distance of oilseed rape; (3) 17 oilseed rape fields were within 1–15 m distance of the study grasslands; and (4) 17 oilseed rape fields were isolated by at least 570 m from calcareous grasslands (mean distance ± SE: 1,598 ± 59.7 m; ESM 2). Each of the 17 study grasslands of category 2 was in 1–15 m distance of one of the studied oilseed rape fields of category 3. Study grasslands and oilseed rape fields were at least 1 km apart from other study grasslands and oilseed rape fields, respectively. At 300 m distance from calcareous grassland, abundances of cavity-nesting bees have been shown to be reduced by 95 % compared to grasslands (Krewenka et al. 2011). All study sites where we recorded *Osmia bicornis* in our traps were included in the analyses of pollen contents and brood cell numbers (in all cases >9 brood cells per site): (1) 14 grasslands isolated from oilseed rape; (2) 12 grasslands adjacent to oilseed rape; and (3) 10 oilseed rape fields adjacent to grasslands. Category 4 (oilseed rape fields isolated from grasslands) was excluded from the analyses of pollen contents and brood cell numbers because only 2 sites had been colonized by *O. bicornis*. Instead, the 34 oilseed rape fields of category 3 and 4 were included in an analysis of *O. bicornis* incidence (see below).

Oilseed rape fields were sown with *Brassica napus* in the autumn of the previous year and were conventionally managed with usually one insecticide application during the flowering period. Isolated grasslands and grasslands adjacent to oilseed rape were similar in management, exposition, inclination and size (grasslands isolated from oilseed rape: mean size ± SE: 1.7 ± 0.3 ha, min: 0.2 ha, max: 6.8 ha; grasslands adjacent to oilseed rape: mean size ± SE: 1.7 ± 0.5 ha, min: 0.1 ha, max: 4.8 ha). Flower cover (% cover of flower corollas per area ground surface) and the number of plant species flowering were recorded once during oilseed rape flowering in a 0.1-ha plot per grassland, and did not significantly differ (all *P* > 0.12) between grasslands isolated from oilseed rape (flower cover: 0.10 ± 0.07 %, min: 0.0001 %, max: 1.0 %; species number: 4.07 ± 0.58, min: 1, max: 9) and grasslands adjacent to oilseed rape (flower cover: 0.46 ± 0.17 %, min: 0.0001 %, max: 2.12 %; species number: 4.58 ± 0.51, min: 3, max: 7).

Around each study site, oilseed rape fields and semi-natural habitats (calcareous grasslands, orchard meadows, old fallows, hedgerows) were mapped in landscape circles with radii of 250, 500, 750, and 1,000 m. The proportions of oilseed rape fields in the landscape circles were calculated with GIS software (ESRI ArcView 3.2). The proportion of oilseed rape spanned a gradient from 0 to 65, 29, 23, and 17 % in the 250, 500, 750, and 1,000 m radius, respectively, and was not correlated with any other habitat type (Spearman rank correlations, all *P* > 0.1, *n* = 34), but was highly negatively correlated with the distance to the next oilseed rape field at all scales (Spearman rank correlations, all *P* < 0.001, *n* = 34). The proportion of semi-natural habitats spanned a gradient from 0.3 to 43, 26, 15, and 13 %, respectively, and was positively correlated with the Shannon Index of habitat diversity in landscape circles with 750 and 1,000 m radius (Spearman rank correlations, all *P* < 0.01, *n* = 33).

### Pollen and brood cells in trap nests

We established trap nests in the edge and the center of our 67 study sites to assess the effect of oilseed rape on pollen-collecting behavior and the number of brood cells of *O. bicornis*. This solitary, polylectic bee can nest in a variety of naturally pre-existing cavities and also colonizes artificial trap nests. *O. bicornis* is the most abundant cavity-nesting bee in the study area (Holzschuh et al. 2010). Between April and June, females of *O. bicornis* build nests with up to 30 brood cells (Westrich 1989) and collect pollen for larval provisioning nearby their nests within a radius of up to 600 m (Gathmann and Tscharntke 2002). A high number of brood cells per trap nest can reflect both a preference of females to nest at this specific location and a high reproductive output per female.

Trap nests consisted of four plastic tubes (20 cm long, 10.5 cm diameter), each filled with about 200 internodes (20 cm long) of common reed *Phragmites australis* (see Tscharntke et al. 1998) and fixed on a post at a height of 1.2 m. Internodes which contain brood cells of *O. bicornis* can be easily recognised by a clay cap, which the bee builds at the end of the internode. One female can occupy more than one internode. We placed trap nests in the centre and the edge of the 67 study sites (134 trap nests with 536 plastic tubes). In oilseed rape fields, edge trap nests were placed between the first and the second row of oilseed rape plants, center trap nests were placed at 20 m distance from the edge. In grasslands, edge trap nests were placed at 1 m distance from the habitat border and center trap nests were placed at 20 m distance from the edge trap nests. Trap nests were established in March and checked for *O. bicornis* nests at the beginning of oilseed rape flowering in April. No nests had been built by *O.bicornis* before the beginning of oilseed rape flowering. Directly after the end of oilseed rape flowering (26 days later in May), all reed internodes containing bee brood cells were collected, stored at 4 °C to stop larval development, and opened in the laboratory. The number of *O. bicornis* brood cells per trap was recorded (Tscharntke et al. 1998).

For the identification of forage plants, pollen was collected from the first and the last brood cell per reed internode. These two most separated brood cells within a reed internode are normally built with an interval of several days inbetween (Westrich 1989) and might thus mirror the pollen-collection behavior at two different time points during oilseed rape flowering. All brood cells of *O. bicornis* contained large amounts of pollen, because larval development had not made considerable progress by that time. After transferring a small sample of the pollen from the brood cell to a glass slide, the percentage pollen of Brassicaceae was determined under a light microscope for 300 pollen grains, which were located in a randomly chosen cluster within the sample. We assume that all pollen of the Brassicaceae pollen came from oilseed rape, because no other Brassicaceae were abundantly flowering at that time in grasslands, crop fields, or field margin strips. Pollen analyses were conducted on 843 brood cells from the 36 sites where *O. bicornis* occurred. The numbers of brood cells were summed over the four plastic tubes of a trap nest per post and the two trap nests per site, and the percentage oilseed rape pollen per brood cell was averaged over all brood cells of a site, because the position of the trap nests in the field neither affected the number of brood cells (*t* test; *F*_1,55_ = 1.3, *P* = 0.25) nor the percentage oilseed rape pollen (*t* test; *F*_1,55_ = 1.4, *P* = 0.24).

### Statistical analyses

The effect of grassland on the incidence (presence or absence) of *O. bicornis* in oilseed rape was assessed in a generalized linear model with quasibinomial errors and the predictor presence of adjacent grassland (oilseed rape adjacent to grassland vs. oilseed rape isolated from grassland).

To assess whether the number of *O. bicornis* brood cells in grasslands is higher adjacent to oilseed rape than isolated from oilseed rape and increases with the proportion of oilseed rape in the landscape, we performed ANCOVAs (type I sums of squares) with the dependent variable number of *O. bicornis* brood cells per grassland and the predictors presence of adjacent oilseed rape (grassland adjacent to oilseed rape vs. grassland isolated from oilseed rape), proportion of oilseed rape in the surrounding landscape circle and their interaction. Separate models were calculated for the different landscape circles (250, 500, 750, 1,000 m radius).

The effects of local and landscape-scale availability of oilseed rape pollen on the percentage oilseed rape pollen in larval food were assessed in ANCOVAs with percentage oilseed rape pollen as the dependent variable and the predictor site type (oilseed rape adjacent to grassland vs. grassland adjacent to oilseed rape vs. grassland isolated from oilseed rape) and their interaction. Separate models were calculated for the different landscape circles (250, 500, 750, 1,000 m radius). In the pollen models, we considered the 36 sites where trap nests had been colonized by *O. bicornis*. These were 14 of the 16 sites of category 1 (grasslands isolated from oilseed rape) and 22 of 34 sites of categories 2 and 3 (grasslands adjacent to oilseed rape and oilseed rape adjacent to grassland). All colonized sites of category 1 and eight colonized sites of categories 2 and 3 were spatially separated from all other sites; in seven cases, the two sites of categories 2 and 3 were directly adjacent to each other. For these seven cases, we additionally conducted a *t* test for paired samples (sites of category 2 vs. 3) and compared the result to the result of the ANCOVA to check whether neglecting the partly nested structure of the model affected the outcome of the ANCOVA model. Furthermore, we assessed—for grasslands isolated from oilseed rape—the relationship between percentage oilseed rape pollen in larval food and the distance from the nearest oilseed rape field in a linear regression model.

To assess whether the number of *O. bicornis* brood cells increased with increasing percentage of oilseed rape pollen in larval food, we calculated linear regression models with the dependent variable number of brood cells per site and the predictor percentage of oilseed rape pollen in larval food. Again, we considered those 36 sites where trap nests had been colonized by *O. bicornis*. The hypothesis that the positive impact of oilseed rape pollen decreases with increasing availability of alternative food resources at the local and landscape scale was tested in separate linear regression models for grasslands and oilseed rape fields with the dependent variable number of brood cells per site, the predictors percentage of oilseed rape pollen, proportion of semi-natural habitats in the surrounding landscape circle, flower cover, and diversity of flowering plants in the grassland (or in the adjacent grassland in case of oilseed rape fields), and the two-fold interactions between percentage oilseed rape pollen and the other predictors. Separate models were calculated for the four landscape circles. All models were computed in R (v.2.11.1; R Development Core Team 2011). Maximal models were simplified in a manual stepwise backward selection on the basis of *F* tests (Crawley 2007). Predictors with *p* > 0.05 were removed from the maximal models. Tukey’s post hoc test for multiple comparisons of means were performed with heteroscedastic consistent covariance estimation, which is a robust method for comparing means of groups with unbalanced group sizes (Herberich et al. 2010; packages multcomp and sandwich).

## Results

We recorded 2,473 brood cells in nests of *Osmia bicornis*, 1,104 brood cells in 12 grasslands adjacent to oilseed rape, 715 brood cells in 14 grasslands isolated from oilseed rape, 606 brood cells in 10 oilseed rape fields adjacent to grasslands, and 48 brood cells in 2 oilseed rape fields isolated from grassland. Trap nests were not colonized by *O. bicornis* in 8 of 34 grasslands and 22 of 34 oilseed rape fields.

**Fig. 1:**
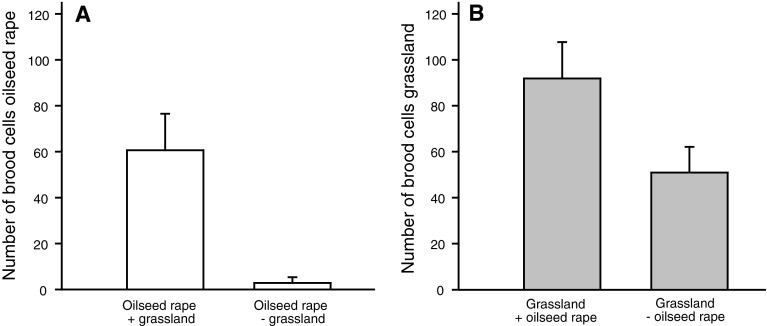
The mean number (±SE) of Red Mason Bee *Osmia bicornis* brood cells in trap nests in **a** oilseed rape fields adjacent to grassland (oilseed rape + grassland; *n* = 17) versus isolated oilseed rape fields (oilseed rape − grassland; *n* = 17; model with presence–absence data), **b** grasslands adjacent to oilseed rape (grassland + oilseed rape; *n* = 17) versus isolated grasslands (grassland − oilseed rape; *n* = 16; model with number of brood cell data)

The presence of adjacent grassland had a positive effect on the number of brood cells in oilseed rape resulting in 606 brood cells in nests of the *O. bicornis* in 10 of 17 oilseed rape fields adjacent to grassland (59 % of the fields), but only 48 brood cells in 2 of 17 oilseed rape fields isolated from grassland (12 % of the fields; GLM with quasibinomial errors: *F*_1,32_ = 8.3, *P* = 0.007; Fig. 1a). The presence of adjacent oilseed rape had a positive effect on the number of brood cells in grasslands. The mean number of brood cells per site was 55 % higher in grasslands adjacent to oilseed rape than in isolated grasslands (Fig. 1b; *R* ^2^ = 0.16, *F*_1,24_ = 4.6, *P* = 0.041), but was not affected by the proportion of oilseed rape in the landscape at any of the four spatial scales.

**Fig. 2:**
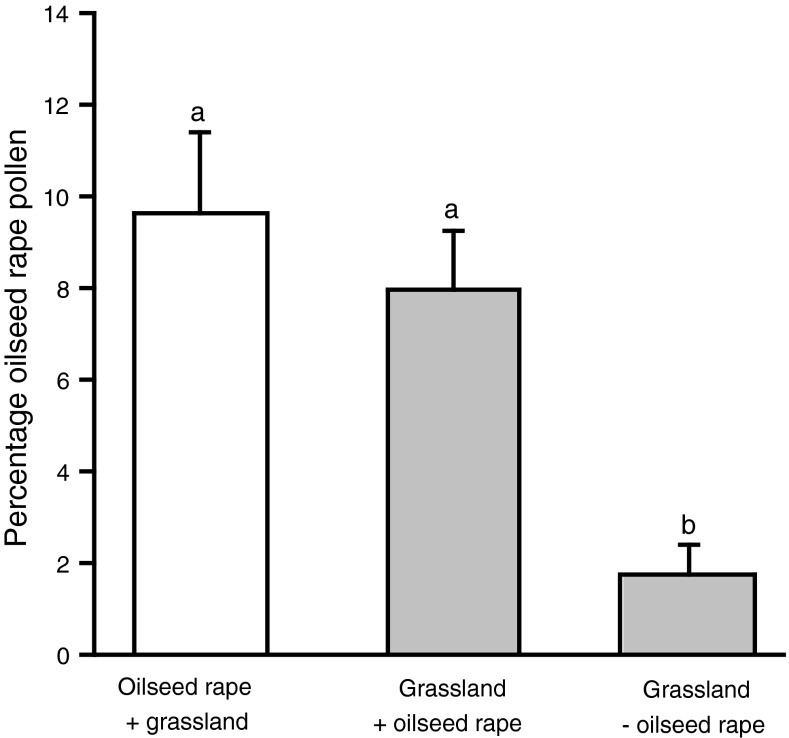
The mean (± SE) percentage oilseed rape pollen in larval food of *O. bicornis* in oilseed rape fields adjacent to grassland (oilseed rape + grassland; *n* = 17), grasslands adjacent to oilseed rape (grassland + oilseed rape; *n* = 17) and isolated grasslands (grassland − oilseed rape; *n* = 16). Data from isolated oilseed rape field were not analyzed, because only 2 of 17 fields had been colonized by *O. bicornis.* Different *letters* indicate significant differences (*P* < 0.05; Tukey contrasts)

The percentage oilseed rape pollen in larval food was higher in oilseed rape fields (*t* = 4.0, *P* < 0.001) and in grasslands adjacent to oilseed rape (*t* = 4.1, *P* < 0.001) than in grasslands isolated from oilseed rape, but did not differ between grasslands adjacent to oilseed rape and oilseed rape fields (*t* = 0.7, *P* > 0.7; Fig. 2) and was not affected by the proportion of oilseed rape in the landscape at any of the four spatial scales. Grasslands adjacent to oilseed rape did not differ from directly adjacent oilseed rape fields when analysed in a *t* test for paired samples (*t* = 0.23, *P* > 0.8). The percentage oilseed rape pollen was not related to the distance from the nearest oilseed rape fields in grasslands isolated from oilseed rape (*F* = 0.14, *P* > 0.7), but it was very low in the three most isolated grasslands (745, 850, and 1,000 m apart from the next oilseed rape field) with 0.6, 0.6, and 0 %, respectively. At 655 m distance from the next oilseed rape field, bees still collected 1.4 % oilseed rape pollen, and at 614 m, the maximum percentage (8.5 %) for grasslands isolated from oilseed rape was even found.

**Fig. 3:**
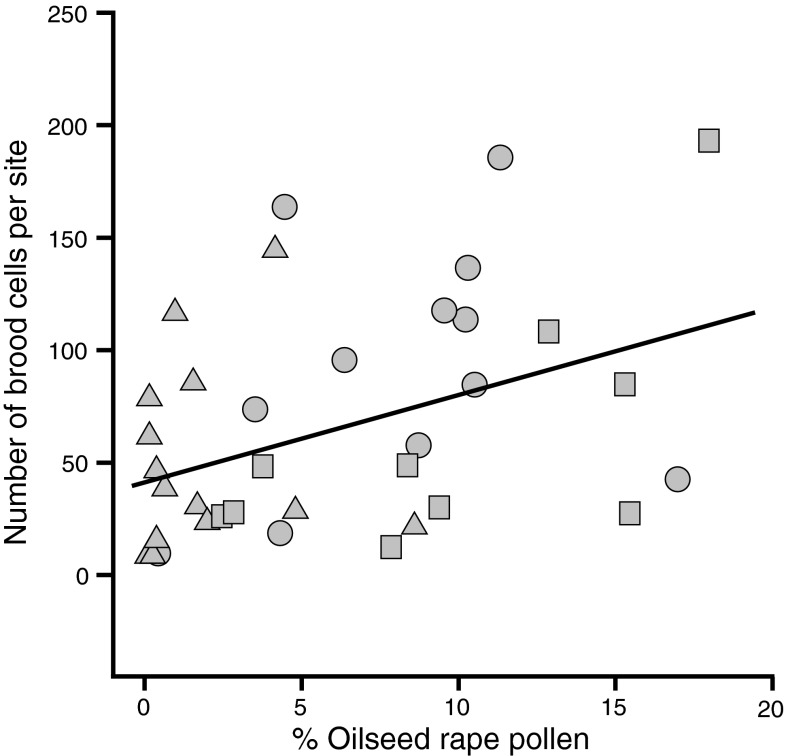
Relationship between the percentage oilseed rape pollen and the number of *O. bicornis* brood cells in oilseed rape fields adjacent to grassland (*squares*), grassland adjacent to oilseed rape (*rounded crackel*), and grassland isolated from oilseed rape (*triangles*). Data from oilseed rape isolated from grassland were not analyzed because only 2 of 17 fields had been colonized by *O. bicornis*

The number of *O. bicornis* brood cells increased with increasing percentage of oilseed rape pollen in the larval food (Fig. 3; *R* ^2^ = 0.16, slope = 3.9; *F*_1,34_ = 6.5, *P* = 0.015). The number of brood cells was not affected by local or landscape-scale availability of alternative food resources (measured as flower cover and number of flowering plant species in the grassland/the adjacent grassland, and the proportion of semi-natural habitats in the landscape) or its interactions with percentage oilseed rape pollen.

## Discussion

We found that mass-flowering oilseed rape fields and semi-natural habitats interacted via dispersing and foraging bees, and affected abundances of bees in both habitat types. The probability that the solitary *O. bicornis* colonized trap nests in oilseed rape increased from 12 to 59 % when grassland was adjacent. In grasslands adjacent to oilseed rape fields, the number of brood cells of *O. bicornis* was 55 % higher than in isolated grasslands. The amount of oilseed rape at the landscape scale had no effects. This is in contrast to findings from bumblebees (Westphal et al. 2006; Diekötter et al. 2010; Holzschuh et al. 2011) and from solitary bees (Jauker et al. 2012), where abundances were affected by the amount of oilseed rape in the landscape. However, the results from our study, which was the first study comparing local- and landscape-scale effects of mass-flowering crops in one study setup, suggest that small-scale effects of oilseed rape are much stronger than landscape-scale effects—at least for solitary bees, which perceive their environment at smaller scales than most bumblebees (Westphal et al. 2006; Zurbuchen et al. 2010. Holzschuh et al. 2011). And while at the landscape scale large amounts of mass-flowering crops not only enhance bumblebee abundances (Westphal et al. 2006), but can also reduce them (Diekötter et al. 2010, Holzschuh et al. 2011), at the local scale, mass-flowering crops have so far been found to have only positive effects, namely by attracting bumblebees not only to the crop flowers, but also to wild flowers in the vicinity (Hanley et al. 2011). Our study now shows that the vicinity of oilseed rape also increases the number of brood cells in trap nests. This increase in the number of brood can either result from a higher reproductive output per female and/or from a higher number of females nesting in the trap nest. Although an increase in the number of brood cells per site has been interpreted as an increase of the bees’ reproductive output by Jauker et al. (2012), it might also result from an increase in the number of females preferably nesting in the vicinity of oilseed rape. Only a genetic maternity test or permanent observations of the trap nest assigning occupied reed internodes to (uniquely marked) females would enable us to definitely distinguish between higher reproduction and higher colonization in the vicinity of oilseed rape.

We found that the percentage of oilseed rape pollen in the larval food was higher in oilseed rape fields and in grasslands adjacent to oilseed rape compared to isolated grasslands. Our data suggest that the percentage of oilseed rape pollen in larval food drastically drop at a distance between 614 and 745 m from the next oilseed rape. This is in agreement with data from Gathmann and Tscharntke (2002), which suggest maximum foraging distances of about 600 m. In both oilseed rape fields and grasslands, the number of brood cells increased with increasing percentage of oilseed rape pollen in the larval food. A higher percentage of oilseed rape pollen in the larval food may indicate that *O. bicornis* females had to invest less time in collecting the larval food and therefore could produce more brood cells than those females not collecting oilseed rape pollen. Klein et al. (2004) showed that foraging trip durations of a trap-nesting megachilid bee declined and the number of brood cells marginally increased when the blossom cover in the habitat patch increased. Similarly, Zurbuchen et al. (2010) found that the number of brood cells of a specialized bee decreased when the distance to its only food plant increased.

Despite the obvious advantages of collecting oilseed rape pollen when oilseed rape was adjacent, the mean of percentage oilseed rape pollen in the larval food per site never exceeded 20 %. Although larvae of *O. bicornis* raised on pure oilseed rape pollen successfully developed into adults (Konrad et al. 2008), a combination of multiple plant species might be more beneficial (Roulston and Cane 2000). *O. bicornis* could have been forced to collect pollen from other plants for various reasons: either because the amount of pollen provided by oilseed rape might have declined over the flowering period thus making collecting oilseed rape pollen less efficient, or because the composition of amino acid or other nutritional components of oilseed rape was unfavorable (Roulston and Cane 2000; Cook et al. 2003), or because pesticide residues might have repelled bees (Thompson 2003), or, finally, because of toxic oilseed rape pollen compounds (Sedivy et al. 2011). Oilseed rape contains all amino acids essential for honey bees (Weiner et al. 2010); however, the ability to digest pollen of a certain plant species varies strongly even among closely related polylectic bee species and within bee populations (Sedivy et al. 2011). Our study shows that the number of brood cells increased with the percentage of oilseed rape pollen in the larval food up to a percentage of 20 %. However, our data do not reveal larval mortality or the reproductive success of the next generation. Further studies are required to assess the effects of oilseed rape pollen on bee fitness in the following generation.

## Conclusion

Our study shows that oilseed rape enhances the abundance of a generalist solitary bee in nearby habitats, and that the number of bee brood cells increased with increasing percentage of oilseed rape pollen in larval food. Our results might be representitive for a large number of bee species, because the majority of European bees are food generalists (Westrich 1989) and might be able to visit oilseed rape. A precondition for the positive effect of oilseed rape is that natural nesting sites (e.g., in semi-natural grassland) are directly adjacent to the oilseed rape field. Thus, an increase of the amount of oilseed rape in the landscape can only be expected to promote wild bees if nesting habitats are already present or if the amount of nesting habitat increases simultaneously with the amount of oilseed rape.

We can only speculate about the longer-term consequences of oilseed rape in the vicinity of semi-natural habitats. Oilseed rape may increase competition among cavity-nesting bee species where nesting sites are the most limiting resource (Steffan-Dewenter and Schiele 2008). This effect could favor early generalist bee species, which could pre-empt nesting cavities for species with more specialized pollen requirements or with later phenology. Furthermore, this effect is likely to carry over into the next season, when a high number of brood cells in grasslands adjacent to oilseed rape in one year will result in a high number of emerging adults in the subsequent year. Thus, competitive pressure on bees that do not benefit from oilseed rape will increase in the subsequent year—regardless of the presence of oilseed rape in the subsequent year. *O. bicornis* is already the most abundant solitary bee in Central Europe (Westrich 1989). Oilseed rape might cause that particularly generalist species that are already dominant might become even more competitive, resulting in negative effects on rare and endangered species in semi-natural or natural habitats.

Wild bees that visit oilseed rape fields might enhance farmers’ yield, because seed set of oilseed rape depends at least partly on bee pollination (Morandin and Winston 2005; Hoyle et al. 2007). In contrast, wild plants might suffer from a lack of pollination when pollinators prefer foraging in oilseed rape instead of visiting wild plants (Holzschuh et al. 2011). The reproductive success of wild plants flowering simultaneously with oilseed rape and mainly pollinated by generalist bees might also be reduced by deposition of rape pollen on wild flowers. On the other hand, the higher number of emerging adults in the year after oilseed rape flowering might compensate for the lack of pollinators in the previous year and reduce potential pollination limitation of wild plants. Further studies are needed to assess the effects of oilseed rape on competition for pollinators between crops versus wild plants, and on competition for nesting sites between pollinators benefiting from oilseed rape versus non-benefiting pollinators. These studies should take short-term effects (during vs. after mass-flowering period) into account as well as long-term effects via crop rotations that result in annual changes in the distribution of mass-flowering crop fields.

## Electronic supplementary material

Below is the link to the electronic supplementary material.

Supplementary material 1 (DOC 1680 kb)

Supplementary material 2 (DOC 35 kb)
