# Supplementary material for: Mass-flowering crops enhance wild bee abundance
Source: Oecologia. 2012 Nov 1;172(2):477–84. doi: 10.1007/s00442-012-2515-5 (PMC3655217; doi:10.1007/s00442-012-2515-5)
Supplement: Supplementary file 2 — Supplementary material 2 (DOC 35 kb) [file 442_2012_2515_MOESM2_ESM.doc]

“Mass-flowering crops enhance wild bee abundance” by Andrea Holzschuh, Carsten F. Dormann, Teja Tscharntke, Ingolf Steffan-Dewenter

**ESM 2: Study design.**
